# The Association Between Depressive Symptoms and Limitations in Physical Functioning

**DOI:** 10.1155/da/9990793

**Published:** 2025-11-10

**Authors:** Shakila Meshkat, Qiaowei Lin, Vanessa K. Tassone, Hilary Y. M. Pang, Wendy Lou, Venkat Bhat

**Affiliations:** ^1^Interventional Psychiatry Program, St. Michael's Hospital, Unity Health Toronto, Toronto, Ontario, Canada; ^2^Department of Psychiatry, University of Toronto, Toronto, Ontario, Canada; ^3^Department of Biostatistics, Dalla Lana School of Public Health, University of Toronto, Toronto, Ontario, Canada; ^4^Institute of Medical Science, Temerty Faculty of Medicine, University of Toronto, Toronto, Ontario, Canada; ^5^Neuroscience Research Program, St. Michael's Hospital, Toronto, Ontario, Canada

**Keywords:** depressive symptoms, function, NHANES, physical function

## Abstract

**Background:**

Depression, a prevalent mood disorder, is often accompanied by considerable functional impairment. Its relationship with specific physical functioning domains and potential variations by symptom type or sex, however, has not been fully clarified. This study investigates these associations, paying particular attention to overall severity, sex differences, and cognitive–affective versus somatic symptom dimensions.

**Methods:**

Data were drawn from 31,336 adults who participated in the 2005–2018 National Health and Nutrition Examination Survey (NHANES). Depressive symptoms were measured using the Mental Health–Depression Screener (Patient Health Questionnaire [PHQ-9]), while functional outcomes were based on the Physical Functioning questionnaire (PFQ). Multivariable logistic regression models were applied to examine associations.

**Results:**

Of all participants, 2534 (7.09%) met criteria for depressive symptoms. These individuals demonstrated markedly greater odds of functional limitations across multiple domains. The strongest association was observed for limitations in leisure and social activities (LSAs; aOR = 8.04; 95% CI = 6.68–9.67), while the weakest was for lower extremity mobility (LEM; aOR = 4.51; 95% CI = 3.53–5.76). Each incremental increase in PHQ-9 score was linked to higher odds of limitations, including 21% greater odds for LSAs (95% CI = 1.19–1.24) and 16% for LEM (95% CI = 1.14–1.19). Sex-stratified analyses suggested that females reported fewer impairments in activities of daily living (ADLs), instrumental ADLs (IADLs), LEM, and general physical activities (GPAs) than males. Cognitive–affective and somatic subscales both showed the strongest associations with LSAs (aOR = 1.35; 95% CI = 1.30–1.40; and aOR = 1.40; 95% CI = 1.37–1.44, respectively) and the weakest with LEM (aOR = 1.25; 95% CI = 1.21–1.29; and aOR = 1.32; 95% CI = 1.27–1.37, respectively).

**Conclusions:**

Depressive symptoms are consistently and strongly related to physical functioning difficulties, with variations across symptom domains and sex. Future studies should explore underlying mechanisms and validate these findings.

## 1. Introduction

Major depressive disorder (MDD) is a persistent mood condition affecting 7.1% of individuals in the United States (US) each year [1]. Rather than presenting as a uniform condition, MDD comprises two sets of symptoms—somatic and cognitive–affective [2]. Research suggests that analyzing these clusters individually provides a more nuanced understanding of the disorder than evaluating overall depression scores alone [3]. Symptoms within these domains can lead to considerable functional impairment and commonly include enduring sadness, diminished interest or pleasure in activities, significant alterations in appetite or body weight, sleep disruption, fatigue, and intense feelings of guilt or worthlessness [4]. These manifestations extend beyond psychological distress, exerting measurable effects on overall physical well-being [5].

Depression ranks among the foremost contributors to global disability, significantly influencing the overall burden of disease [6]. The functional limitations observed in depression are thought to arise from disruptions in emotional, cognitive, and physical domains [7, 8]. These impairments manifest in various ways, such as diminished capacity to manage activities of daily living (ADLs), impaired social interactions, and decreased ability to perform work or school tasks effectively [8]. Importantly, depression can affect individuals' ability to perform ADLs, leading to limitations in routine physical functioning [9]. Evidence indicates that, even after accounting for coexisting medical conditions, depression independently impairs both role performance and physical functioning [10]. This means that individuals with MDD often experience limitations in their physical capabilities, which can exacerbate feelings of helplessness and further contribute to the overall disability burden [11].

Because physical functioning underpins daily living and quality of life, disruptions in this domain among individuals with depression can have far-reaching health implications. Physical functioning includes the capacity to carry out daily activities, participate in physical exercise, and maintain autonomy—factors that are essential for overall well-being and quality of life [12, 13]. However, the association of depression and physical functioning is not unidirectional. While depression can impair physical functioning, physical limitations themselves may also increase the risk of developing or exacerbating depressive symptoms [13]. This reciprocal relationship suggests a dynamic interplay, where physical and mental health challenges reinforce one another.

While the relevance of physical functioning to overall well-being is well established, how it intersects with depression is less clear. In particular, the possibility that impaired physical capacity may both result from and contribute to depressive symptoms has received limited attention [14, 15], leaving a gap in understanding how these processes mutually influence one another. To advance theoretical understanding, it is essential to establish hypotheses that account for the bidirectional nature of these associations. In addition, it is unclear how depression is associated with the various domains of physical functioning or whether there are any potential sex differences. Understanding how depressive symptoms relate to physical functioning, as well as whether these relationships vary between males and females, is crucial for tailoring effective interventions. Accordingly, this study aims to examine the links between depressive symptoms—including overall severity and specific symptom clusters—and physical functioning, while also exploring potential sex-based differences in these associations.

## 2. Methods

### 2.1. Study Sample

We analyzed data from the 2005–2018 cycles of the National Health and Nutrition Examination Survey (NHANES), a nationally representative, cross-sectional survey. NHANES is administered by the National Center for Health Statistics (NCHS) at the Centers for Disease Control and Prevention and is a key source of information for population health research in the US. NHANES employs a stratified, multilevel probability sampling approach to ensure representation of the US civilian, noninstitutionalized population. Participants provide informed consent under protocols approved by the NCHS Ethics Review Board. Information is gathered through home interviews covering sociodemographic characteristics, health-related behaviors, and dietary practices, supplemented by standardized physical assessments and laboratory testing at mobile examination centers. This design allows detailed profiling of the US population's health and nutrition.

For this study, we included respondents who completed the Physical Functioning questionnaire (PFQ; the related questions we examined were only administered to adults aged 20 years and above) and the Mental Health—Depression Screener questionnaire (DPQ). CDC website represents more details on NHANES methodology (https://wwwn.cdc.gov/nchs/nhanes/analyticguidelines.aspx).

### 2.2. Exposure Variable

Depressive symptoms were determined with the nine-item Patient Health Questionnaire-9 (PHQ-9), a self-reported tool widely adopted in psychiatric and psychological research. The scale, aligned with DSM-5 diagnostic criteria, covers key domains including mood, interest, sleep, appetite, concentration, energy, feelings of worthlessness, psychomotor changes, and suicidal thoughts [16]. Respondents rate how often each symptom was present during the past 2 weeks on a 0–3 scale (0 = “not at all,” 3 = “nearly every day”). A cumulative score of 10 or more is generally interpreted as reflecting clinically meaningful depressive symptoms, while lower scores suggest minimal or absent symptoms [17, 18]. The PHQ-9's items can also be separated into two subdomains: cognitive–affective (items 1, 2, 6, 7, 9) and somatic (items 3, 4, 5, 8), which are analyzed as continuous variables. Prior work has consistently demonstrated the PHQ-9's brevity, strong psychometric reliability, and broad applicability across both clinical and research settings [16–18].

### 2.3. Outcome Variable

Physical functioning was assessed with the PFQ during in-home interviews administered through a computer-assisted format. Participants indicated whether they could carry out 17 different physical tasks without specialized equipment, which can be grouped into five functional domains (Table S1) [19]: (1) ADLs; (2) instrumental ADLs (IADLs); (3) leisure and social activities (LSAs); (4) lower extremity mobility (LEM); and (5) general physical activities (GPAs). Response options included “no difficulty,” “some difficulty,” “much difficulty,” or “unable to do.” Individuals who indicated any level of difficulty or inability with at least one task within a domain were considered to have functional limitations in that area. Conversely, participants who reported no difficulty in all tasks corresponding to specific domains, or who responded “No” to questions about limitations keeping them from working, experiencing confusion or memory problems, physical, mental, or emotional limitations, and requiring special healthcare equipment were considered without limitation.

### 2.4. Covariates

To minimize potential confounding, the analysis adjusted for several covariates, including age (continuous), sex (male/female), race/ethnicity (Mexican American, other Hispanic, non-Hispanic White, non-Hispanic Black, and other/multiracial), family income-to-poverty ratio (PIR) (classified into two groups: low (≤1.3) and mid-to-high (>1.3)). Educational attainment was categorized into four levels: less than high school, high school or equivalent, some college/associate degree, and college graduate or higher. Body mass index (BMI) was categorized as underweight (<18.5 kg/m^2^), normal weight (18.5–24.9 kg/m^2^), overweight (25–29.9 kg/m^2^), and obese (≥30 kg/m^2^) [20]. Marital status was coded as never married, married, widowed, divorced, separated, and living with a partner.

Medical conditions were identified from questionnaire responses. Diabetes was defined by a “yes” response to the item asking whether a doctor had ever diagnosed them with diabetes (excluding pregnancy-related cases); those answering “no” or “borderline” were considered nondiabetic. Hypertension was established from affirmative answers to questions about ever being diagnosed with high blood pressure or being told on at least two occasions they had hypertension. Stroke and coronary heart disease (CHD) were classified based on self-reported doctor diagnoses, and cancer was defined as a self-reported history of any malignancy.

### 2.5. Statistical Analysis

All analysis procedures use the survey package in R (v4.4.0) to incorporate the complex NHANES sampling design, which accounts for clustering, oversampling, nonresponse, and poststratification. Survey weights were applied and divided by the total number of survey waves (seven in this analysis) in accordance with NHANES guidelines. Because each survey wave consisted of a different group of participants, the combined dataset was treated as a cross-sectional sample for analysis. Descriptive statistics accounted for the survey design and weights. Differences in depressive symptom status (depressed vs. not depressed) were examined with *t*-tests for continuous outcomes and chi-square tests for categorical outcomes, with statistical significance determined at *p*  < 0.05.

Associations between depressive symptoms (binary classification), PHQ-9 total scores, and the cognitive–affective and somatic subscales with each physical functioning domain were analyzed using multivariable logistic regression models. To evaluate potential sex-specific effects, interaction terms for sex were included. Additional exploratory analyses reversed the exposure and outcome framework, treating physical functioning domains as predictors and depressive measures as outcomes. In the adjusted models, all predefined covariates were included, whereas unadjusted models considered only the primary exposures. Missing data was handled using a complete-case approach.

## 3. Results

### 3.1. Sample Characteristics

**Figure 1:**
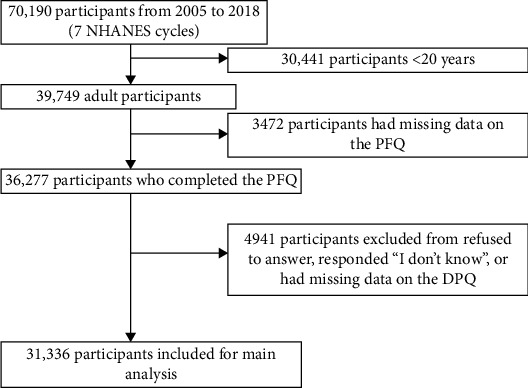
Participant inclusion flowchart. DPQ, Mental Health - Depression Screener Questionnaire; NHANES, National Health and Nutrition Examination Survey; PFQ, Physical Functioning Questionnaire.

Data from 70,190 participants were collected across seven NHANES cycles from 2005 to 2018 (Figure 1). The final study population comprised 31,336 individuals, among whom 2534 (7.09%) had depressive symptoms, 3113 (7.59%) reported limitations in ADLs, 4167 (10.96%) reported limitations in IADLs, 3366 (8.67%) reported limitations in LSAs, 4848 (12.23%) reported limitations in LEM, and 7114 (19.21%) reported limitations in GPAs.

The average age of the study population was 46.6 years (SD = 16.6), and just over half (51.29%) were female. The cohort was ethnically diverse, comprising 8.50% Mexican American, 10.93% non-Hispanic Black, and 67.77% non-Hispanic White participants. Most individuals had some college education or higher (64.67%), and 79.83% were classified as having mid-to-high income. Regarding marital status, over half (55.80%) were married. Approximately 28.33% were classified as having a healthy BMI, while 36.97% were obese. The prevalence of chronic conditions was 8.68% for diabetes, 24.63% for hypertension, 2.31% for stroke, 2.99% for CHD, and 9.66% for cancer.

A comprehensive overview of participant demographic and clinical characteristics, together with statistically significant covariate differences, is provided in Table 1.

### 3.2. Depressive Symptoms and Physical Functioning

**Figure 2:**
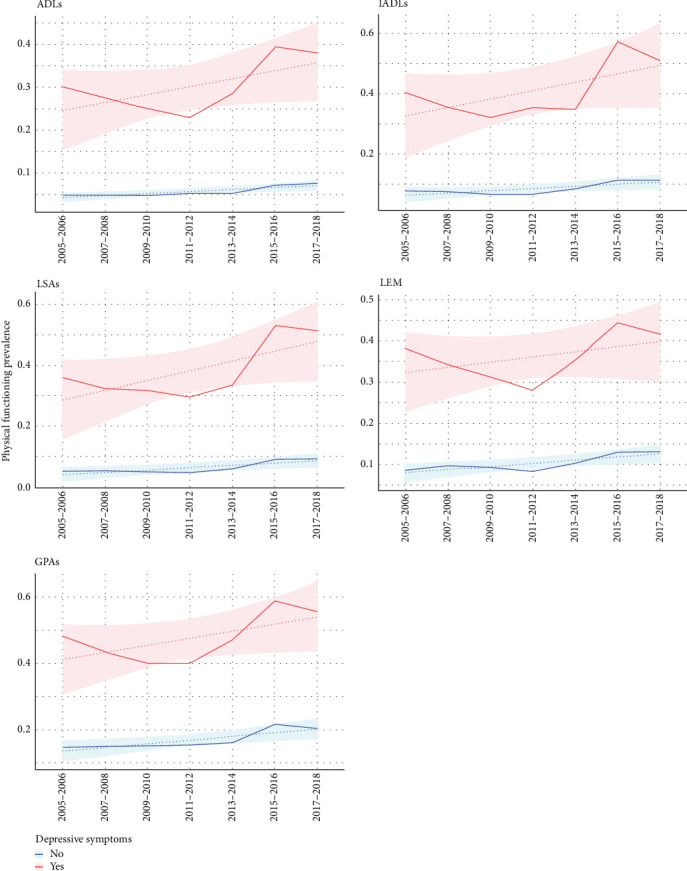
Mean trajectory of limitations in physical functioning prevalence by depressive symptoms.

The analysis revealed strong associations between depressive symptoms and functional limitations across multiple domains (Table 2, Figure 2). Compared with individuals without depressive symptoms, those with symptoms were more likely to experience limitations: 6.36 times greater odds for ADLs (95% confidence interval [CI] = 5.06–8.00), 6.65 times for IADLs (95% CI = 5.25–8.43), 8.04 times for LSAs (95% CI = 6.68–9.67), 4.51 times for LEM (95% CI = 3.53–5.76), and 4.93 times for GPAs (95% CI = 4.03–6.02).

Moreover, each one-point rise in PHQ-9 score corresponded to higher odds of functional limitations: a 19% increase for ADLs (95% CI = 1.17–1.22), 20% for IADLs (95% CI = 1.17–1.22), 21% for LSAs (95% CI = 1.19–1.24), 16% for LEM (95% CI = 1.14–1.19), and 17% for GPAs (95% CI = 1.15–1.19).

Additional results (Table S3) showed that greater physical functioning limitations were also significantly associated with greater odds and severity of depressive symptoms.

### 3.3. Interaction Effects of Sex and Depressive Symptoms on Physical Functioning

The interaction effects suggested that sex modified how depressive symptoms related to physical functioning, with significant interaction effects observed for ADLs, IADLs, LEM, and GPAs (*p*  < 0.05), where females experienced less limitations in physical functioning compared to males (aOR < 1, Table 3). Stratified analyses (Table S2) further supported this finding, indicating that depressive symptoms were more strongly linked to functioning impairments in males (adjusted odds ratio [aOR] range: 6.76–10.01) than in females (aOR range: 3.63–7.05). Similarly, increases in PHQ-9 scores were more strongly associated with higher odds of physical functioning limitations in males (aOR range: 1.19–1.23) than in females (aOR range: 1.15–1.17).

### 3.4. Cognitive–Affective and Somatic Scores and Physical Functioning

The analyses showed that both cognitive–affective and somatic scores were significantly linked to physical functioning limitations across multiple domains (Table 4). Increases in cognitive–affective scores were consistently related to higher odds of functional impairments: a 31% for ADLs (95% CI = 1.27–1.35), 33% for IADLs (95% CI = 1.28–1.38), 35% for LSAs (95% CI = 1.30–1.40), 25% for LEM (95% CI = 1.21, 1.29), and 27% for GPAs (95% CI = 1.23–1.31).

Somatic scores showed stronger associations, with each one-point increase corresponding to 32% to 40% higher odds of limitations across physical functioning domains.

## 4. Discussion

Our findings indicated a robust relationship between depressive symptoms and impairments across multiple domains of physical functioning. Higher levels of depressive symptoms were linked to greater odds of limitations in ADLs, IADLs, LSAs, LEM, and GPAs. Participants reporting depressive symptoms were significantly more likely to experience these functional limitations than those without such symptoms. It is important to emphasize that, due to the cross-sectional design, these results do not establish causality; the direction of effect—whether depressive symptoms contribute to functional impairments or vice versa—remains unclear. These statistical associations highlight the need for longitudinal or experimental studies to clarify causal pathways. Sex differences were observed in some domains: females showed lower odds of limitations in ADLs, IADLs, LEM, and GPAs compared to males, whereas no significant sex difference was noted for LSAs. Both cognitive–affective and somatic symptom clusters were associated with greater odds of functional limitations across all domains. These findings support theoretical models proposing a reciprocal relationship between depressive symptoms and physical functioning, in which depressive symptoms may reduce functional capacity, while functional impairments may, in turn, exacerbate depressive symptoms through mechanisms such as diminished independence, heightened stress, or social withdrawal. Future research employing longitudinal designs will be critical to examining these bidirectional processes.

Our results align with prior research that indicated the link between depression and functional impairment [21–23]. Furthermore, the stronger association between somatic, rather than affective, symptoms and physical functioning was expected due to the direct influence of physical symptoms, such as fatigue and pain, on daily activities, which more directly limit an individual's ability to perform routine tasks [2–4]. The stronger association with depressive symptoms was observed for LSAs, followed by IADLs. The high odds ratio for limitations in LSAs suggests that depression impairs an individual's social and leisure engagement, which may lead to increased social isolation and reduced quality of life. This significant association may be due to the combination of physical lethargy, lack of motivation, and social withdrawal commonly associated with depressive symptoms. Following closely, IADLs include tasks that are supportive of independent living, such as housekeeping and meal preparation. The increased odds ratio in this domain suggests that depression compromises the cognitive and executive functioning required to perform these complex daily tasks. This can lead to increased reliance on others, further diminishing the individual's sense of autonomy and self-efficacy. The domain that was most weakly associated with depressive symptoms was LEM. LEM encompasses the ability to move physically and manage emotional responses effectively. While still significant, the relatively lower odds ratio in this domain compared to others may be suggestive of the inherent variability in how depression affects physical and emotional responses. Some individuals with depression may still retain a degree of physical mobility and emotional regulation, possibly due to variability in the severity of symptoms or the presence of effective coping mechanisms. The differential association across domains highlights the complex interplay between depression and functional ability. The severe limitations in LSAs and IADLs suggest that depression predominantly affects activities requiring higher levels of cognitive engagement and social interaction. In contrast, the relatively lower association with LEM indicates that basic physical movement and emotional regulation, while affected, might be more resilient or less uniformly impaired by depressive symptoms. These observations emphasize the need for future research to investigate how depressive symptom clusters interact with various domains of physical functioning over time, incorporating a theoretical framework that accounts for bidirectional effects.

Our findings on sex differences in the link between depressive symptoms and physical functioning domains were inconsistent with previous studies, which either found no significant differences between sexes [24] or reported greater impairment in females [25]. This discrepancy may be due to several factors. Variations in sample characteristics, such as age, socioeconomic status, and overall health, could influence the outcomes, as different populations may exhibit varying degrees of vulnerability to depressive symptoms and their association with physical functioning. Additionally, the measurement tools used to assess depressive symptoms and physical functioning might differ between studies, with some utilizing different scales or criteria that could affect the results. One possible explanation for why our study found relatively lower impairment in females could be sex differences in the expression of depression; females tend to report emotional and psychological symptoms more readily and seek help, while men might underreport these symptoms and exhibit depression through externalizing behaviors like substance abuse or aggression, leading to greater functional impairment [26–29]. Additionally, social and cultural factors may influence the perceived and actual impact of depression, as societal expectations often differ for men and females, potentially affecting how each sex experiences and reports functional impairment [26–29]. Finally, the stigma associated with mental health issues might be more pronounced for men, causing them to delay seeking help and thus suffer more severe consequences in their daily functioning [26–29].

While the NHANES dataset utilized in this study offers significant strengths in its rigorous sampling design and comprehensive data collection methods, several limitations should be considered. First, the cross-sectional design of NHANES precludes conclusions about causality between depressive symptoms and physical functioning. The study relied on self-reported measures, including the PHQ-9 for depressive symptoms and the PFQ for physical functioning, which may be influenced by recall bias or social desirability. Despite adjusting for multiple covariates, residual confounding is possible due to unmeasured factors or inaccuracies in self-reported variables such as BMI and medical history. Furthermore, while NHANES captures US nationally representative samples, certain groups—such as institutionalized individuals and those without stable housing—are excluded, which may affect the applicability of these findings to other populations. These factors underscore the need for careful interpretation and for future research in more diverse and specific populations.

In conclusion, this study highlights a robust association between depressive symptoms and limitations in physical functioning, emphasizing the interconnected nature of mental and physical health. The results suggest that depressive symptoms are linked to impaired daily functioning and underscore the importance of integrated approaches in clinical and public health practice. Observed sex differences point to the potential benefit of tailored interventions that address varying vulnerabilities in physical functioning among individuals with depression. Moving forward, longitudinal research is essential to clarify the temporal relationships between depressive symptoms and functional limitations and to explore underlying biological and psychosocial mechanisms. Such insights could guide targeted interventions to prevent functional decline and enhance quality of life. Incorporating assessments of physical functioning into routine mental health care may further improve treatment outcomes and contribute to more effective strategies for managing both depressive symptoms and associated disability.

## Figures and Tables

**Table 1:** Participant characteristics by depressive symptom status.

Characteristic	Total	Depressive symptoms no	Depressive symptoms yes	*p*-Value
Sample size	31,336	28,802	2534	—
PHQ-9 total scores (mean [SD])	2.89 (3.92)	2.05 (2.34)	13.95 (3.73)	<0.001
ADLs = yes (%)	3113 (7.59)	2234 (5.85)	879 (30.39)	<0.001
IADLs = yes (%)	4167 (10.96)	3048 (8.66)	1119 (41.15)	<0.001
LSAs = yes (%)	3366 (8.67)	2331 (6.39)	1035 (38.61)	<0.001
LEM = yes (%)	4848 (12.23)	3778 (10.40)	1070 (36.20)	<0.001
GPAs = yes (%)	7114 (19.21)	5779 (17.02)	1335 (47.87)	<0.001
Age (mean [SD])	46.55 (16.63)	46.63 (16.70)	45.58 (15.62)	0.027
Sex = female (%)	16,080 (51.29)	14,447 (50.29)	1633 (64.37)	<0.001
Race (%)				
Mexican American	4978 (8.50)	4593 (8.52)	385 (8.16)	<0.001
Non-Hispanic black	6696 (10.93)	6128 (10.73)	568 (13.52)	—
Non-Hispanic white	13,283 (67.77)	12,214 (68.08)	1069 (63.71)	—
Other Hispanic	2966 (5.46)	2648 (5.31)	318 (7.43)	—
Other Race—Including Multiracial	3413 (7.35)	3219 (7.37)	194 (7.17)	—
PIR = mid-to-high income (%)	20,131 (79.83)	19,008 (81.41)	1123 (59.25)	<0.001
Education (%)				
Less than high school	3554 (12.88)	3116 (12.26)	438 (20.56)	<0.001
High school graduate/GED	3955 (22.46)	3598 (22.10)	357 (26.92)	—
Some college/AA degree	5599 (32.26)	5127 (31.98)	472 (35.65)	—
College graduate or above	4657 (32.41)	4474 (33.66)	183 (16.88)	—
BMI (%)				
Healthy weight	8461 (28.33)	7884 (28.58)	577 (25.05)	<0.001
Underweight	475 (1.53)	434 (1.51)	41 (1.83)	—
Overweight	10,366 (33.16)	9702 (33.65)	664 (26.81)	—
Obese	11,782 (36.97)	10,562 (36.26)	1220 (46.31)	—
Marital status (%)				
Never married	5839 (18.33)	5283 (18.00)	556 (22.65)	<0.001
Married	16,279 (55.80)	15,393 (57.23)	886 (36.99)	—
Widowed	2169 (4.98)	1933 (4.80)	236 (7.38)	—
Divorced	3334 (10.10)	2899 (9.59)	435 (16.84)	—
Separated	1023 (2.32)	857 (2.09)	166 (5.28)	—
Living with partner	2676 (8.47)	2422 (8.29)	254 (10.87)	—
Diabetes = yes (%)	3644 (8.68)	3198 (8.32)	446 (13.37)	<0.001
HTN = yes (%)	8556 (24.63)	7566 (23.77)	990 (35.90)	<0.001
Stroke = yes (%)	951 (2.31)	779 (2.05)	172 (5.62)	<0.001
CHD = yes (%)	1086 (2.99)	937 (2.84)	149 (5.01)	<0.001
Cancer = yes (%)	2765 (9.66)	2486 (9.53)	279 (11.32)	0.053

*Note*: Categorical variables are presented as unweighted counts with weighted percentages. Continuous variables are expressed as weighted means with standard deviations. Statistical significance was determined at p < 0.05 for comparisons by depressive symptom status.

Abbreviations: ADLs: activities of daily living; BMI: body mass index; CHD: coronary heart disease; GPAs: general physical activities; HTN: hypertension; IADLs: instrumental activities of daily living; LEM: lower extremity mobility; LSAs: leisure and social activities; PHQ-9: patient health questionnaire-9; PIR: family income-to-poverty ratio; SD: standard deviation.

**Table 2:** Associations between depressive symptoms (presence and PHQ-9 scores) and physical functioning outcomes.

Depressive symptoms—yes				
Physical functioning domain	OR (95% CI)	*p*-Value	aOR (95% CI)	*p*-Value
ADLs	7.03 (6.20,7.96)	**<0.001**	6.36 (5.06,8.00)	**<0.001**
IADLs	7.38 (6.56,8.30)	**<0.001**	6.65 (5.25,8.43)	**<0.001**
LSAs	9.22 (8.31,10.23)	**<0.001**	8.04 (6.68,9.67)	**<0.001**
LEM	4.89 (4.36,5.48)	**<0.001**	4.51 (3.53,5.76)	**<0.001**
GPAs	4.47 (4.04,4.95)	**<0.001**	4.93 (4.03,6.02)	**<0.001**

**PHQ-9 total scores**				
**Physical functioning domain**	**OR** **(95% CI)**	***p***-**Value**	**aOR** **(95% CI)**	***p***-**Value**

ADLs	1.19 (1.18,1.20)	**<0.001**	1.19 (1.17,1.22)	**<0.001**
IADLs	1.20 (1.19,1.22)	**<0.001**	1.20 (1.17,1.22)	**<0.001**
LSAs	1.22 (1.21,1.23)	**<0.001**	1.21 (1.19,1.24)	**<0.001**
LEM	1.16 (1.15,1.17)	**<0.001**	1.16 (1.14,1.19)	**<0.001**
GPAs	1.15 (1.14,1.16)	**<0.001**	1.17 (1.15,1.19)	**<0.001**

*Note*: The reference level for depressive symptoms is “no.” Statistical significance was determined at *p* < 0.05. Bold values are statistically significant.

Abbreviations: aOR, adjusted odds ratio; CI, confidence interval.

**Table 3:** Interaction effects of sex and depressive symptoms (presence and PHQ-9 total scores) on physical functioning outcomes.

Depressive symptoms—yes sex—female				
Physical functioning domain	OR (95% CI)	*p*-Value	aOR (95% CI)	*p*-Value
ADLs	0.67 (0.52,0.86)	**0.002**	0.58 (0.36,0.95)	**0.031**
IADLs	0.54 (0.41,0.71)	**<0.001**	0.49 (0.31,0.77)	**0.003**
LSAs	0.69 (0.53,0.90)	**0.006**	0.70 (0.44,1.10)	0.119
LEM	0.62 (0.50,0.77)	**<0.001**	0.52 (0.35,0.77)	**0.002**
GPAs	0.59 (0.47,0.75)	**<0.001**	0.44 (0.31,0.64)	**<0.001**

**PHQ-9 total scores** **sex—Female**				
**Physical functioning domain**	**OR** **(95% CI)**	***p***-**Value**	**aOR** **(95% CI)**	***p***-**Value**

ADLs	0.97 (0.95,0.99)	**0.003**	0.97 (0.94,1.00)	**0.030**
IADLs	0.96 (0.94,0.98)	**<0.001**	0.96 (0.92,0.99)	**0.018**
LSAs	0.98 (0.96,1.00)	**0.022**	0.99 (0.96,1.02)	0.359
LEM	0.97 (0.95,0.99)	**0.001**	0.96 (0.94,0.99)	**0.015**
GPAs	0.97 (0.95,0.99)	**0.001**	0.96 (0.93,0.99)	**0.006**

*Note*: The reference level for sex is “male”; the reference level for depressive symptoms is “no”. Statistical significance was determined at *p* < 0.05. Bold values are statistically significant.

Abbreviations: aOR, adjusted odds ratio; CI, confidence interval.

**Table 4:** Associations of PHQ-9 cognitive–affective and somatic components with physical functioning outcomes.

Cognitive–affective scores				
Physical functioning domain	OR (95% CI)	*p*-Value	aOR (95% CI)	*p*-Value
ADLs	1.31 (1.29,1.34)	**<0.001**	1.31 (1.27,1.35)	**<0.001**
IADLs	1.35 (1.32,1.37)	**<0.001**	1.33 (1.28,1.38)	**<0.001**
LSAs	1.37 (1.35,1.40)	**<0.001**	1.35 (1.30,1.40)	**<0.001**
LEM	1.26 (1.24,1.28)	**<0.001**	1.25 (1.21,1.29)	**<0.001**
GPAs	1.24 (1.22,1.26)	**<0.001**	1.27 (1.23,1.31)	**<0.001**

**Somatic scores**				
**Physical functioning domain**	**OR** **(95% CI)**	***p***-**Value**	**aOR** **(95% CI)**	***p***-**Value**

ADLs	1.38 (1.35,1.40)	**<0.001**	1.38 (1.33,1.43)	**<0.001**
IADLs	1.38 (1.36,1.40)	**<0.001**	1.36 (1.32,1.41)	**<0.001**
LSAs	1.42 (1.40,1.44)	**<0.001**	1.40 (1.37,1.44)	**<0.001**
LEM	1.31 (1.29,1.34)	**<0.001**	1.32 (1.27,1.37)	**<0.001**
GPAs	1.28 (1.27,1.30)	**<0.001**	1.33 (1.29,1.37)	**<0.001**

*Note:* Statistical significance was determined at *p* < 0.05. Bold values are statistically significant.

Abbreviations: aOR, adjusted odds ratio; CI, confidence interval.

## Data Availability

Data for this study can be publicly accessed via https://www.cdc.gov/nchs/nhanes/index.htm. The statistical analysis plan and analytic code that supports the findings of this study are available from the corresponding author upon reasonable request.

## Nomenclature

PHQ-9: Patient Health Questionnaire-9
ADLs: Activities of daily living
IADLs: Instrumental activities of daily living
LSAs: Leisure and social activities
LEM: Lower extremity mobility
GPAs: General physical activities
PIR: Family income-to-poverty ratio
BMI: Body mass index
HTN: Hypertension
CHD: Coronary heart disease
SD: Standard deviation.

## Supporting Information

Supporting Information Table S1. Five domains of physical functioning. Table S2. Association between depressive symptoms severity (PHQ-9 scores)/depressive symptoms and physical functioning stratified by sex. Table S3. Regression models examining associations of physical functioning with presence of depressive symptoms and depressive symptom severity (PHQ-9 scores) (Outcome: depressive symptoms).

## References

[1] Zhdanava M., Pilon D., Ghelerter I. (2021). The Prevalence and National Burden of Treatment-Resistant Depression and Major Depressive Disorder in the United States. *The Journal of Clinical Psychiatry*.

[2] Elhai J. D., Contractor A. A., Tamburrino M. (2012). The Factor Structure of Major Depression Symptoms: A Test of Four Competing Models Using the Patient Health Questionnaire-9. *Psychiatry Research*.

[3] Hoen P. W., Whooley M. A., Martens E. J., Na B., van Melle J. P., de Jonge P. (2010). Differential Associations Between Specific Depressive Symptoms and Cardiovascular Prognosis in Patients With Stable Coronary Heart Disease. *Journal of the American College of Cardiology*.

[4] Li Z., Ruan M., Chen J., Fang Y. (2021). Major Depressive Disorder: Advances in Neuroscience Research and Translational Applications. *Neuroscience Bulletin*.

[5] Jones H. J., Minarik P. A., Gilliss C. L., Lee K. A. (2020). Depressive Symptoms Associated With Physical Health Problems in Midlife Women: A Longitudinal Study. *Journal of Affective Disorders*.

[6] Friedrich M. J. (2017). Depression Is the Leading Cause of Disability Around the World. *JAMA*.

[7] Spitzer R. L., Kroenke K., Linzer M. (1995). Health-Related Quality of Life in Primary Care Patients With Mental Disorders. Results From the PRIME-MD 1000 Study. *JAMA*.

[8] Kessler R. C., Berglund P., Demler O. (2003). The Epidemiology of Major Depressive Disorder: Results From the National Comorbidity Survey Replication. *JAMA*.

[9] Paterson D. H., Warburton D. E. R. (2010). Physical Activity and Functional Limitations in Older Adults: A Systematic Review Related to Canada’s Physical Activity Guidelines. *International Journal of Behavioral Nutrition and Physical Activity*.

[10] Mossey J. M., Gallagher R. M., Tirumalasetti F. (2000). The Effects of Pain and Depression on Physical Functioning in Elderly Residents of a Continuing Care Retirement Community. *Pain Medicine*.

[11] Callahan C. M., Wolinsky F. D., Stump T. E. (1998). Mortality, Symptoms, and Functional Impairment in Late-Life Depression. *Journal of General Internal Medicine*.

[12] Painter P., Stewart A. L., Carey S. (1999). Physical Functioning: Definitions, Measurement, and Expectations. *Advances in Renal Replacement Therapy*.

[13] Garatachea N., Molinero O., Martínez-García R., Jiménez-Jiménez R., González-Gallego J., Márquez S. (2009). Feelings of Well Being in Elderly People: Relationship to Physical Activity and Physical Function. *Archives of Gerontology and Geriatrics*.

[14] Ormel J., Rijsdijk F. V., Sullivan M., Van Sonderen E., Kempen G. I. (2002). Temporal and Reciprocal Relationship Between IADL/ADL Disability and Depressive Symptoms in Late Life. *The Journals of Gerontology Series B: Psychological Sciences and Social Sciences*.

[15] Ames M. E., Robillard C. L., Ryan J. E. H., Merrin G. J., Turner B. J. (2023). Reciprocal Associations Between Physical Activity, Physical Self-Concept, Somatic Symptoms, and Depression From Adolescence to Young Adulthood: Disaggregating Within-and Between-Person Effects. *Mental Health and Physical Activity*.

[16] Kroenke K., Spitzer R. L., Williams J. B. (2001). The PHQ-9: Validity of a Brief Depression Severity Measure. *Journal of General Internal Medicine*.

[17] Kroenke K., Spitzer R. L. (2002). The PHQ-9: A New Depression Diagnostic and Severity Measure. *Psychiatric Annals*.

[18] Costantini L., Pasquarella C., Odone A. (2021). Screening for Depression in Primary Care With Patient Health Questionnaire-9 (PHQ-9): A Systematic Review. *Journal of Affective Disorders*.

[19] Cosiano M. F., Jannat-Khah D., Lin F. R., Goyal P., McKee M., Sterling M. R. (2020). Hearing Loss and Physical Functioning Among Adults With Heart failure: Data From NHANES. *Clinical Interventions in Aging*.

[20] Weir C. B., Jan A. (2025). *BMI Classification Percentile and Cut off Points*.

[21] Hammer-Helmich L., Haro J. M., Jönsson B. (2018). Functional Impairment in Patients With Major Depressive Disorder: The 2-Year PERFORM Study. *Neuropsychiatric Disease and Treatment*.

[22] Nagar S., Sherer J. T., Chen H., Aparasu R. R. (2010). Extent of Functional Impairment in Children and Adolescents With Depression. *Current Medical Research and Opinion*.

[23] Ito M., Bentley K. H., Oe Y. (2015). Assessing Depression Related Severity and Functional Impairment: The Overall Depression Severity and Impairment Scale (ODSIS). *PLoS ONE*.

[24] Carmona N. E., Subramaniapillai M., Mansur R. B. (2018). Sex Differences in the Mediators of Functional Disability in Major Depressive Disorder. *Journal of Psychiatric Research*.

[25] Sloan D. M., Sandt A. R. (2006). Gender Differences in Depression. *Women’s Health*.

[26] Albert P. R. (2015). Why Is Depression More Prevalent in Women?. *Journal of Psychiatry and Neuroscience*.

[27] Miller K., Greyling M., Cooper C., Lu L., Sparks K., Spector P. E. (2000). Occupational Stress and Gender: A Cross-Cultural Study. *Stress Medicine*.

[28] Slavich G. M., Sacher J. (2019). Stress, Sex Hormones, Inflammation, and Major Depressive Disorder: Extending Social Signal Transduction Theory of Depression to Account for Sex Differences in Mood Disorders. *Psychopharmacology*.

[29] Padesky C. A., Hammen C. L. (1981). Sex Differences in Depressive Symptom Expression and Help-Seeking Among College Students. *Sex Roles*.

