# Supplementary material for: The Association Between Depressive Symptoms and Limitations in Physical Functioning
Source: Depress Anxiety. 2025 Nov 10;2025:9990793. doi: 10.1155/da/9990793 (PMC12623077; doi:10.1155/da/9990793)
Supplement: Supporting Information — Table S1. Five domains of physical functioning. Table S2. Association between depressive symptoms severity (PHQ-9 scores)/depressive symptoms and physical functioning stratified by sex. Table S3. Regression models examining associations of physical functioning with presence of depressive symptoms and depressive symptom severity (PHQ-9 scores) (Outcome: depressive symptoms). [file 9990793.f1.docx]

**Supplementary Materials**

**Table S1.**

| **Domains of Physical Functioning** | **Specific Physical Tasks Within Each Domain** |
| --- | --- |
| Activities of Daily Living (ADLs) | Getting in and out of bed |
|  | Using fork, knife, cup |
|  | Dressing yourself |
| Instrumental Activities of Daily Living (IADLs) | Managing money |
|  | Doing house chores |
|  | Preparing meals |
| Leisure and Social Activities (LSAs) | Going out to movies/events |
|  | Attending social events |
|  | Home leisure activities |
| Lower Extremity Mobility (LEM) | Walking for a quarter mile |
|  | Walking up ten stairs |
|  | Walking between rooms |
| General Physical Activities (GPAs) | Lifting or carrying |
|  | Standing for long periods |
|  | Sitting for long periods |
|  | Reaching up over head |
|  | Grasp/holding small objects |

**Table S2.**

| **PHQ-9 Scores (Female Models)** | | | | |
| --- | --- | --- | --- | --- |
| Physical functioning Domain | OR  (95% CI) | *p*-value | aOR  (95% CI) | *p*-value |
| ADLs | 1.18  (1.16,1.19) | **<0.001** | 1.17  (1.14,1.20) | **<0.001** |
| IADLs | 1.18  (1.17,1.20) | **<0.001** | 1.18  (1.15,1.21) | **<0.001** |
| LSAs | 1.21  (1.19,1.22) | **<0.001** | 1.21  (1.18,1.23) | **<0.001** |
| LEM | 1.14  (1.13,1.16) | **<0.001** | 1.15  (1.12,1.18) | **<0.001** |
| GPAs | 1.13  (1.12,1.14) | **<0.001** | 1.15  (1.13,1.18) | **<0.001** |
| **PHQ-9 Scores (Male Models)** | | | | |
| Physical functioning Domain | OR  (95% CI) | *p*-value | aOR  (95% CI) | *p*-value |
| ADLs | 1.21  (1.19,1.23) | **<0.001** | 1.23  (1.19,1.26) | **<0.001** |
| IADLs | 1.23  (1.21,1.25) | **<0.001** | 1.23  (1.19,1.26) | **<0.001** |
| LSAs | 1.24  (1.22,1.25) | **<0.001** | 1.22  (1.19,1.25) | **<0.001** |
| LEM | 1.18  (1.16,1.19) | **<0.001** | 1.19  (1.16,1.22) | **<0.001** |
| GPAs | 1.17  (1.15,1.19) | **<0.001** | 1.20  (1.17,1.24) | **<0.001** |
| **Depressive symptoms - yes (Female Models)** | | | | |
| Physical functioning Domain | OR  (95% CI) | *p*-value | aOR  (95% CI) | *p*-value |
| ADLs | 5.94  (5.00,7.05) | **<0.001** | 4.84  (3.70,6.33) | **<0.001** |
| IADLs | 5.67  (4.85,6.63) | **<0.001** | 5.21  (3.94,6.89) | **<0.001** |
| LSAs | 7.81  (6.71,9.10) | **<0.001** | 7.05  (5.60,8.89) | **<0.001** |
| LEM | 3.94  (3.42,4.53) | **<0.001** | 3.63  (2.81,4.69) | **<0.001** |
| GPAs | 3.56  (3.14,4.05) | **<0.001** | 3.72  (3.00,4.63) | **<0.001** |
| **Depressive symptoms - yes (Male Models)** | | | | |
| Physical functioning Domain | OR  (95% CI) | *p*-value | aOR  (95% CI) | *p*-value |
| ADLs | 8.88  (7.39,10.67) | **<0.001** | 9.63  (6.59,14.07) | **<0.001** |
| IADLs | 10.48  (8.57,12.82) | **<0.001** | 10.01  (6.89,14.54) | **<0.001** |
| LSAs | 11.27  (9.42,13.49) | **<0.001** | 9.75  (6.96,13.67) | **<0.001** |
| LEM | 6.34  (5.32,7.56) | **<0.001** | 6.76  (4.67,9.77) | **<0.001** |
| GPAs | 5.99  (5.01,7.17) | **<0.001** | 7.84  (5.57,11.04) | **<0.001** |

**Note: aOR = adjusted odds ratio; CI = confidence interval; the reference level for depressive symptoms is “no”; ADLs = Activities of daily living; IADLs = Instrumental activities of daily living; LSAs = Leisure and social activities; LEM = Lower extremity mobility; GPAs = General physical activities; p-values < 0.05 denote statistical significance.**

**Table S3.**

| **Presence of depressive symptoms** | | | | |
| --- | --- | --- | --- | --- |
| Physical functioning Domain | OR  (95% CI) | *p*-value | aOR  (95% CI) | *p*-value |
| ADLs - yes | 7.03 (6.20,7.96) | **<0.001** | 6.03 (4.76,7.64) | **<0.001** |
| IADLs - yes | 7.38 (6.56,8.30) | **<0.001** | 6.60 (5.17,8.43) | **<0.001** |
| LSAs - yes | 9.22 (8.31,10.23) | **<0.001** | 7.94 (6.56,9.60) | **<0.001** |
| LEM - yes | 4.89 (4.36,5.48) | **<0.001** | 4.16 (3.22,5.37) | **<0.001** |
| GPAs - yes | 4.47 (4.04,4.95) | **<0.001** | 4.86 (3.95,5.98) | **<0.001** |
| **PHQ-9 Scores** | | | | |
| Physical functioning Domain | Coef.  (95% CI) | *p*-value | aCoef.  (95% CI) | *p*-value |
| ADLs - yes | 4.30 (3.99,4.62) | **<0.001** | 4.07 (3.59,4.56) | **<0.001** |
| IADLs - yes | 4.14 (3.87,4.41) | **<0.001** | 3.86 (3.42,4.29) | **<0.001** |
| LSAs - yes | 4.80 (4.54,5.06) | **<0.001** | 4.47 (4.08,4.86) | **<0.001** |
| LEM - yes | 3.19 (2.97,3.41) | **<0.001** | 2.93 (2.54,3.32) | **<0.001** |
| GPAs - yes | 2.63 (2.43,2.83) | **<0.001** | 2.73 (2.39,3.06) | **<0.001** |

**Note: aOR = adjusted odds ratio; aCoef. = adjusted coefficient estimates; CI = confidence interval; ADLs = Activities of daily living; IADLs = Instrumental activities of daily living; LSAs = Leisure and social activities; LEM = Lower extremity mobility; GPAs = General physical activities; the reference level for all physical functioning domain is no; p-values < 0.05 denote statistical significance.**
